# *Two-in-one* strategy for optimizing chemical and structural properties of carbon felt electrodes for vanadium redox flow batteries

**DOI:** 10.1080/14686996.2024.2327274

**Published:** 2024-03-06

**Authors:** Sung Joon Park, Min Joo Hong, Ye Ji Ha, Jeong-In Choi, Ki Jae Kim

**Affiliations:** a Department of Energy Science, Sungkyunkwan University, Suwon, Republic of Korea; b Department of Future Energy Engineering, Sungkyunkwan University, Suwon, Republic of Korea; c Department of Energy Engineering, Konkuk University, Seoul, Republic of Korea; d SKKU Institute of Energy Science and Technology (SIEST), Sungkyunkwan University, Suwon, Republic of Korea

**Keywords:** Vanadium redox flow battery, carbon felt, Ni metal etching, porous structure, robust oxygen functional groups

## Abstract

Vanadium redox flow batteries (VRFBs) have received significant attention for use in large-scale energy storage systems (ESSs) because of their long cycle life, flexible capacity, power design, and safety. However, the poor electrochemical activity of the conventionally used carbon felt electrode results in low energy efficiency of the VRFBs and consequently impedes their commercialization. In this study, a carbon felt (CF) electrode with numerous nanopores and robust oxygen-containing functional groups at its edge sites is designed to improve the electrochemical activity of a carbon felt electrode. To achieve this, Ni metal nanoparticles were initially precipitated on the surface of the CF electrode, followed by etching of the precipitated Ni nanoparticles on the CF electrode using sulfuric acid. The resulting CF electrode had a specific surface area eight times larger than that of the pristine CF electrode. In addition, the oxygen-containing functional groups anchored at the graphite edge sites of the nanopores can act as robust electrocatalysts for VO^2+^/VO_2_^+^ and V^2+^/V^3+^ redox reactions. Consequently, the VRFB cell with the resulting carbon felt electrode can deliver a high energy efficiency of 86.2% at the current density of 60 mA cm^−2^, which is 20% higher than that of the VRFB cell with the conventionally heat-treated CF electrode. Furthermore, the VRFB cell with the resultant carbon felt electrodes showed stable cycling performance with no considerable energy efficiency loss over 200 charge-discharge cycles. In addition, even at a high current density of 160 mA cm^−2^ , the developed carbon felt electrode can achieve an energy efficiency of 70.1%.

## IMPACT STATEMENT

This work reveals the importance of the robust graphite edge-site oxygen functional group and the holey structure of the ET-CF electrode, emphasizing that high VRFB efficiency can be achieved by engineering both the structure and surface properties of the carbon felt electrode.

## GRAPHICAL ABSTRACT

**Figure:**
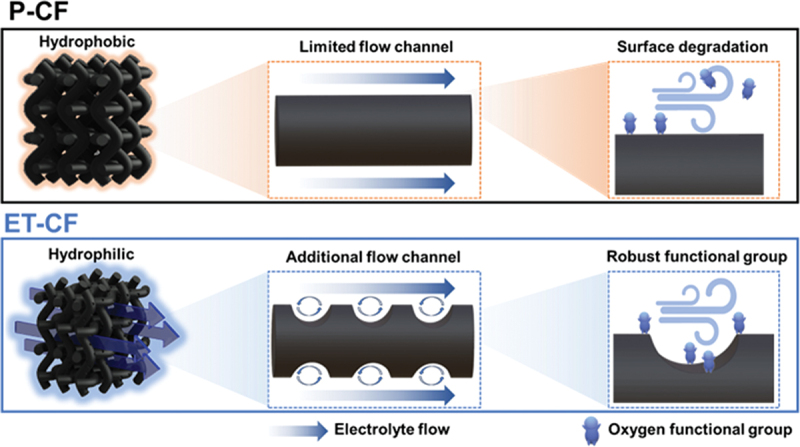

## 1. Introduction

Among various redox flow batteries (RFBs), all vanadium redox flow batteries (VRFBs) have come close to commercialization in large-scale energy storage systems because of their lower cross-contamination by using the same active materials for both catholyte and anolyte, design flexibility, power scalability, high safety, and long cycle life [1–7]. However, their relatively low energy efficiencies are a hurdle for the widespread implementation of VRFBs. Among the various factors affecting the lower energy efficiencies of VRFBs, poor electrochemical activities arising from the hydrophobic nature and restricted surface area of the commonly used carbon felt (CF) electrodes in VRFBs are crucial factors in determining the overall electrochemical performance of VRFB [8–11]. Therefore, the drawbacks of CF electrodes must be addressed for the quick commercialization of VRFBs.

Introducing electrocatalysts such as metal oxides, precious metals, carbon nanotube, and graphene onto the CF electrode has been considered a promising method [12–14]. Although the reported electrocatalysts can improve the electrochemical performance of the CF electrode, the electrocatalysts introduced on the CF electrode are expensive and can be shed from the CF electrode’s surface by fast electrolyte flow. In addition, the polymeric binder used to adhere the electrocatalysts to the CF electrode may resist electron transfer, and the electrocatalysts may induce unwanted side reactions could [15,16]. Therefore, this method appears to be limited in practical applications for VRFB because of the aforementioned shortcomings. Another promising method for improving the electrochemical activity of CF electrodes is to introduce oxygen-containing functional groups onto their surfaces [17,18]. The utilization of oxygen-containing functional groups on CF electrodes has received persistent attention in the research community because of their exceptional ability to enhance the electrochemical performance of VRFBs. Thus, in the past decades, various surface treatments, such as thermal, mild oxidation, acid, and mild acid treatments, have been proposed for the successful introduction of oxygen-containing functional groups [19–24]. However, the electrocatalytic efficacy of oxygen-containing functional groups introduced on the CF electrode still does not satisfy the long-term durability requirements owing to functional group degradation during repeated charge and discharge processes [25,26].

Recent studies have reported that the long-term durability of oxygen-containing functional groups can be ensured by introducing these groups to the edge sites rather than to the basal plane of the CF electrode. They emphasized that oxygen-containing functional groups introduced on the basal plane were more susceptible to degradation during repeated charge and discharge cycling than those at edge sites [24,25]. Many research groups have worked to increase the specific active surface area of CF electrodes by producing pores on their surface using various etching agents such as NiO, ZIF-8, CuO, Co_3_O_4_, and Mn_3_O_4_ [27–31]. They reported that the pores generated on the surface of the CF electrodes not only provide a large active surface area but that the edge sites of the pores can also act as anchor sites for introducing robust functional groups. In addition, they explained that pores can play a role in facilitating mass transport and redox reactions by shortening the diffusion length of vanadium ions [27,28]. Therefore, the pores generated on the surface of the CF electrode are beneficial for increasing the specific surface area and number of edge sites.

By reviewing antecedent studies, it was determined that the most effective way to ensure the excellent and long-lasting electrochemical performance of CF electrodes is to generate numerous edge sites on the surface of the CF electrode and then introduce oxygen-containing functional groups at the edge sites. In response to this perspective, we designed a CF electrode with numerous nanopores and robust oxygen-containing functional groups at their edge sites. To achieve this, Ni metal nanoparticles were initially precipitated onto the surface of the CF electrode, followed by etching the precipitated Ni nanoparticles on the CF electrode using sulfuric acid [32,33]. This facile process not only results in the formation of nanopores on the CF electrode but also allows the incorporation of robust oxygen functional groups onto the edge sites of the nanopores. Consequently, the resulting CF electrode (denoted as ET-CF) has eight times larger specific surface area than the pristine CF electrode (p-CF) by producing numerous nanopores. The oxygen functional groups are robustly anchored at the edge sites of the nanopores, as expected. The significance and efficacy of the oxygen-containing functional groups anchored at the edge sites in the redox reactions of vanadium ions were demonstrated through a comparative study with a heat-treated CF electrode (HT-CF) predominantly featuring basal-plane oxygen-containing functional groups. Owing to these ET-CF electrodes’ unique features, the VRFB cell with the ET-CF electrode exhibited outstanding rate performance at a high current density of 160 mA cm^−2^ and stable long-term cycling performance. This study aims to guide CF electrode design to improve the poor electrochemical activity of CF electrodes toward vanadium redox reactions.

## 2. Materials and methods

### 2.1. Materials

Nickel (II) chloride hexahydrate (NiCl_2_∙6 H_2_O) was obtained from DAEJUNG. Sodium hydroxide (NaOH 98%), acetone (C_3_H_6_O, 99.5%), and sulfuric acid (H_2_SO_4_, 99.5%) were purchased from SAMCHUN. Hydrazine monohydrate (H_2_NNH_2_∙H_2_O, 98^+^%) and methanol anhydrous (CH_3_OH, 99.9%) were obtained from Alfa Aesar. Vanadium (Ⅳ) oxide sulfate hydrate (VOSO_4_, Sigma – Aldrich), carbon felt (CF, XF30A) and Nafion 212 (NR-212, DuPont) membranes were used.

### 2.2. Preparation of nickel-etched carbon felt (ET-CF) electrode and heat-treated carbon felt electrode (HT-CF)

CF electrodes were used for electrochemical measurements. To fabricate the ET-CF, p-CF was stirred in methanol/de-ionized water (1:1 by volume) containing 0.5 g nickel chloride and 0.1 g sodium hydroxide for 30 min to produce nickel metal which was then dried at 60°C for 12 h in a vacuum oven. The electrode was heated at 800°C for 1 h under H_2_/Ar gas flow. The obtained electrode was immersed in sulfuric acid and sonicated for 20 min to completely remove any remaining nickel metal. After acid etching, the electrodes were washed using de-ionized water for neutralization; subsequently, the sample was dried at 60°C for 12 h in vacuum oven. For comparison, pristine carbon felt (p-CF) was heated at 400°C for 4 h under air condition at heating rate of 10°C min^−1^; subsequently, the obtained electrode was washed with acetone using sonication to synthesize HT-CF.

### 2.3. Characterization

The morphologies of p-CF, HT-CF, and ET-CF were studied using field-emission scanning electron microscopy (FE-SEM, SUC8010_EX370 Max50) with a W filament and energy-dispersive X-ray spectroscopy (EDS, SUC8010_EX370 Max50). X-ray diffraction (XRD) data of the bare CF, heat-treated CF, and nickel-etched CF were obtained using X-ray diffractometer (XRD, PANalytical EMPYREAN) with Cu K α radiation (λ = 1.5406 Å). The Raman spectra of the bare CF, heat-treated CF, and Ni-etched carbon felt were verified using a Raman spectrometer (DXR3×i) with a He-Ne laser of 532 nm). The specific surface area was calculated using the Brunauer – Emmett – Teller and Barrett – Joyner – Halenda equations. The contact angle of each sample was determined using the water droplet method and the new Goniostar contact angle software for surface energy and tension measurements. The mechanical strength of each felt sample was estimated using a universal testing machine (UTM, JSV-H1000). The surface chemistry of the electrodes was analyzed using X-ray photoelectron spectroscopy (XPS, K-alpha) with a focused monochromatic Al K-α beam (1486.6 eV).

### 2.4. Electrochemical measurements

Cyclic voltammetry (CV) and electrochemical impedance spectroscopy (EIS) were performed using VMP3 (BioLogic). For CV and EIS, a three-electrode electrochemical cell consisting of an Ag/AgCl reference electrode soaked in 3 M KCl, a platinum wire as the counter electrode, and a CF electrode as the working electrode was used. For CV, 0.01 M VOSO_4_ in 3 M H_2_SO_4_ was used as the catholyte, and 0.01 M V^3+^ in 3 M H_2_SO_4_ was used as the anolyte. CV was performed at various scan rates from 10 to 50 mV s^−1^. EIS was performed at frequencies ranging from 100 to 10 mHz at open-circuit voltages (OCVs).

### 2.5. Single-cell test

The redox flow single cell was composed of electrodes (2 × 3 cm, 6 cm^2^), graphite bipolar plate (BPs_Morgan Korea Co. Ltd.), ion exchange membranes (Nafion 117, Dupont), vanadium electrolyte (1.5 M VOSO_4_ in 3 M H_2_SO_4_ solution), and pumps (LongerPump_YZ1515X). The volumes of both the catholyte and anolyte were 15 mL in each tank. The test cell performance was evaluated using potentiostat/galvanostat (WBC3000, WonATech) in the voltage range of 0.8–1.8 V at a current density of 60–160 mA cm^2^. The flow rate was fixed at 12.34 mL min^−1^ controlled by the pump.

## 3. Result and discussion

### 3.1. Concept proposal of ET-CF

**Scheme 1.**
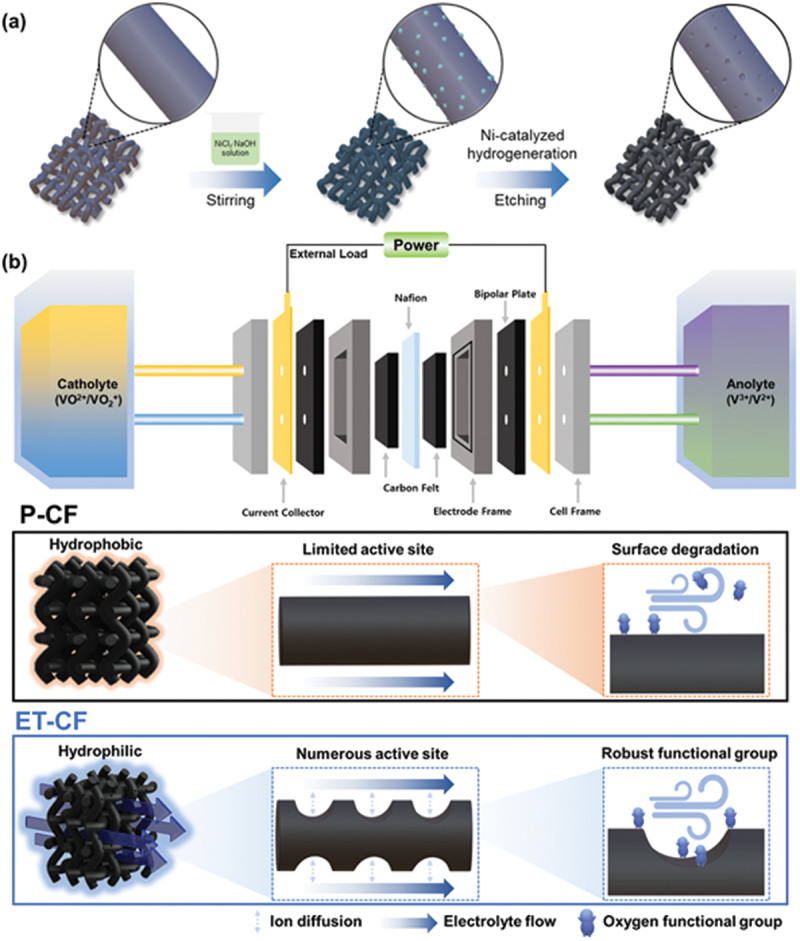
(a) Schematic fabrication process of the preparation process of ET-CF. (b) VRFB single cell schematic with p-CF and ET-CF.

The fabrication process and beneficial properties of ET-CF are shown in Scheme 1. Ni metal was initially incorporated onto the p-CF surface to generate nanosized pores on the CF electrode (Figure. S1). Subsequently, Ni metal was decomposed through a nickel-catalyzed hydrogeneration reaction, resulting in the formation of numerous nanosized pores on the surface of the p-CFs. Finally, an acid treatment step was used to anchor the oxygen functional groups at the edge sites of the nanosized pores and simultaneously remove the unreacted residuals on the p-CF surface (Scheme 1(a)). The resulting EC-CF possesses numerous nanopores with graphite edge-site-anchored oxygen functional groups, which not only facilitate mass transfer but also introduce robust oxygen functional groups that offer active sites for vanadium redox reactions (Scheme 1(b)).

### 3.2. Physicochemical characterization analysis of CF

**Figure 1.**
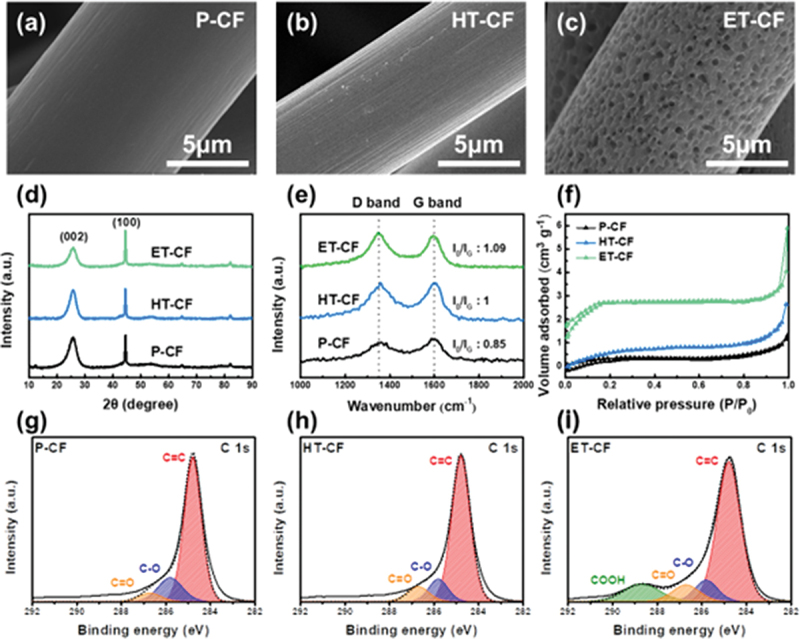
SEM images of (a) p-CF, (b) HT-CF, and (c) ET-CF. (d) XRD pattern, (e) Raman spectra, (f) nitrogen adsorption/desorption isotherm curves of p-CF, HT-CF, ET-CF. XPS C 1s spectra of (g) p-CF, (h) HT-CF, (i) ET-CF.

As shown in Figure 1(a–c), to prove the successful formation of numerous nanosized pores on ET-CF, the surface morphologies of p-CF, HT-CF, and ET-CF were observed by FE-SEM. As expected, uniformly distributed nanosized pores were clearly observed on the surface of ET-CF, whereas a smooth surface was observed on the surface of p-CF. To confirm that the nanopore formation is only caused by Ni metal particles, p-CF without Ni metal particles was heat-treated at 400°C in an air atmosphere. As shown in Figure 1(b), HT-CF has a very smooth surface, indicating that a simple treatment without Ni metal particles cannot form nanopores on the CF surface. Therefore, we confirmed the successful generation of numerous nanopores on the surface of the CF electrode through the implementation of the proposed fabrication process.

The large number of nanopores formed on the surface of the CF electrode might provide a high specific surface area; thus, the increase in the specific surface area was confirmed through N_2_ adsorption/desorption measurements (Figure 1(f) and Figure S2). Even though the tensile strength of ET-CF is slightly reduced compared to that of p-CF owing to the presence of numerous nanopores (Figure S3), which is sufficient to practically apply in VRFB, the specific surface area increased in the order of p-CF < HT-CF < ET-CF (p-CF: 1.33 m^2^ g^−1^, HT-CF: 3.25 m^2^ g^−1^, ET-CF: 10.66 m^2^ g^−1^). In addition, the formation of nanopores on the surface of CF electrodes can cause physical deformation. Thus, to explore the degree of physical deformation of the CF electrode after surface treatment, the crystallinity of each CF electrode was initially investigated using XRD. As shown in Figure 1(d), all samples show two noticeable peaks at 25° and 44°, corresponding to the (002) and (100) crystal planes, respectively [34,35]. Notably, ET-CF shows the smallest (002) peak intensity, indicating that ET-CF exhibits relatively low crystallinity compared to p-CF and HT-CF. This was further confirmed using Raman spectroscopy (Figure 1(e)). Two characteristic peaks located at 1350 and 1600 cm^−2^, attributed to the D band and G band, respectively, showing the disordered and graphitic structure, can be observed in the Raman spectroscopy. The intensity ratio between the D and G bands (I_D_/I_G_) describes the degree of physical deformation; a high intensity ratio indicates the presence of more defects in the CF electrode [26–29]. As shown in Figure 1(e), the I_D_/I_G_ ratio of ET-CF (1.089 for ET) is considerably higher than that of p-CF (0.85), indicating that more graphite edge sites are exposed on the surface of ET-CF through the formation of nanopores.

Because the surface properties of the CF electrodes are crucial factors related to the electrochemical performance of the VRFB, the chemical composition of the surface of the CF electrodes was investigated by XPS and energy-dispersive X-ray spectroscopy analysis, as shown in Figure 1(g–i) and Figure S4–5. After heat and acid treatment, the increased amounts of oxygen functional groups, including C=O (286.7 eV) and COOH (288.8 eV), for HT-CF and ET-CF are consistent with previous studies [36]. Because ET-CF has the largest specific surface area and graphite edge sites, where oxygen groups are prone to attachment, the O/C ratio (Figure S6) is the highest among the three CF electrodes (p-CF: 0.049, HT-CF: 0.137, and ET-CF: 0.279) [27]. Furthermore, the surface chemistry is closely related to the electrolyte affinity, which is one of the most important factors in VRFB application; thus, we conducted an electrolyte droplet test (Figure S7) [27–30]. As ET-CF has the largest number of oxygen functional groups, it shows the smallest contact angle with deionized water, demonstrating that the surface chemistry changed to hydrophilicity after treatment, which is consistent with the results of the XPS analysis. Based on these experimental results, we conjectured that graphite edge sites anchored by oxygen functional groups were formed on a large number of pores using the proposed method.

### 3.3. Electrochemical performance of each CF electrodes on vanadium redox reaction

**Figure 2.**
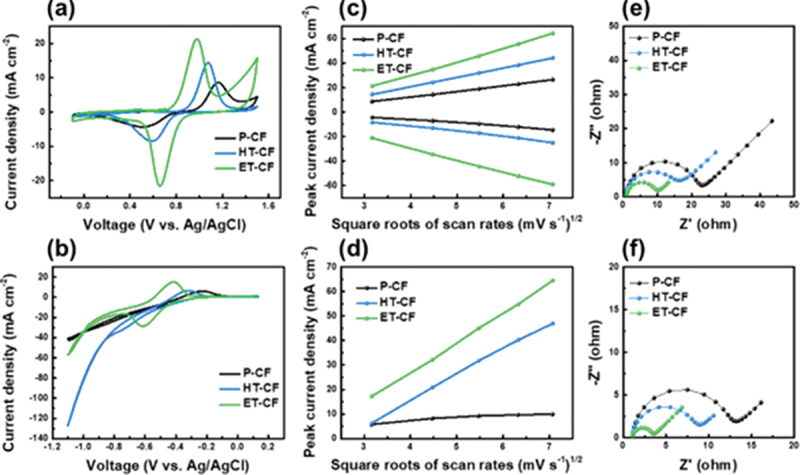
CV curves of (a) VO^2+^/VO_2_^+^ and (b) V^2+^/V^3+^ for p-CF, HT-CF and ET-CF at a scan rate of 10 mV s^−1^. Randles – Sevcik plots of (c) positive peak current density and (d) negative peak current density of p-CF, HT-CF and ET-CF. (e-f) Nyquist plots for p-CF, HT-CF and ET-CF.

The catalytic effect of ET-CF on the vanadium ion redox reaction was evaluated by cyclic voltammetry using a three-electrode cell at room temperature under 0.01 M vanadium ion in a 2 M H_2_SO_4_ condition. As shown in Figure 2(a), the peak potential separation for the VO^2+^/VO_2_^+^ redox reaction (∆E) on ET-CF is 0.32 V, which is much lower than that of p-CF (0.5 V) and HT-CF (0.47 V). The oxidation and reduction peak currents in ET-CF for the VO^2+^/VO_2_^+^ redox reaction in the catholyte were 2.45 and 4.92 times larger than those of p-CF, respectively, indicating that ET-CF shows higher reactivity for the vanadium redox reaction. In addition, ET-CF showed the lowest ratio of the cathodic and anodic peak currents, |I_pa_/I_pc_| measured at the positive electrode (p-CF: 2.38, HT-CF: 1.46, ET-CF: 0.99), indicating that ET-CF exhibits enhanced reversibility for the VO^2+^/VO_2_^+^ redox reaction [17]. For the anolyte solution (Figure 2(b)), in the CV profiles of p-CF and HT-CF, it was difficult to clearly distinguish between the hydrogen evolution reaction (HER) and the reduction of V^3+^ to V^2+^ [37]. In contrast, the ET-CF shows clearly distinct reduction and oxidation peaks related to the V^2+^/V^3+^ redox reaction at −0.6 and −0.42 V, which indicates that the HER and reduction of V^3+^ to V^2+^ are clearly distinguishable. Interestingly, the ratio between the anodic and cathodic peak currents (I_pa_/I_pc_) of ET-CF was 0.53, whereas those of p-CF and HT-CF could not be estimated owing to the asymmetric behavior of the oxidation and reduction reactions. This result implies that ET-CF possesses outstanding reversibility for the V^2+^/V^3+^ redox reactions. Consequently, based on the results of the symmetric oxidation and reduction peaks, redox peak separation, and activity in the catholyte and anolyte, ET-CF showed the highest reactivity and reversibility for vanadium ions originating from the produced graphite edge sites and an expanded specific surface area during the etching treatment, as listed in Table S1. As shown in Figure S8, the long-lasting function of ET-CF was verified by a long-cycle CV test. After the 50^th^ and 100^th^ cycles, ET-CF still exhibited the highest peak current density and lowest overpotential, implying that the graphite edge site-anchored oxygen functional groups on ET-CF possessed outstanding long-term stability. Furthermore, the mass-transport properties were determined using the following equation:

(1) $$\mathrm{Ip}=2.69\times 10^{5}\times AD^{1/2}n^{3/2}\vartheta^{1/2}C$$

where A (cm^2^) represents the active area of the electrode, D (cm^2^ s^−1^) expresses the diffusion coefficient, n denotes the number of electrons participating in the electrochemical reaction, ϑ (V s^−1^) represents the scan rate, and C (mol cm^−3^) represents the concentration of the solution. According to the above equation, the mass transport properties of the CFs can be estimated by fitting the peak current density as a function of the square root of the scan rate (Figure 2(c–d)) [27,28]. ET-CF showed the steepest slope for both the VO^2+^/VO_2_^+^ and V^2+^/V^3+^ redox reactions, implying a significantly increased number of flowing channels in the pores and an expanded specific surface area during the etching process, which facilitated vanadium ion transport (Table S2). The enhanced kinetics and lowered overpotential were further confirmed by the smallest charge transfer resistance of ET-CF for both the catholyte and anolyte vanadium solutions through electrochemical impedance spectroscopy, as plotted in Figure 2(e–f) [18,38]. As a result of the above measurements, because of the facilitated ion diffusivity and decreased charge transfer, ET-CF shows high reversibility and redox activity originating from its robust oxygen-containing functional groups and high specific surface area.

**Figure 3.**
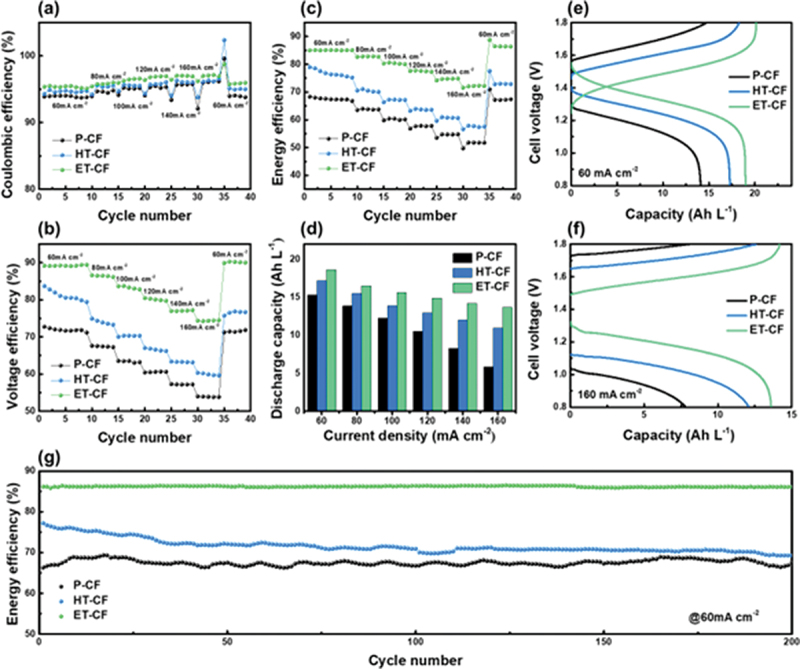
(a) Coulombic efficiencies, (b) voltage efficiencies, (c) energy efficiencies, and (d) discharge capacities at different current densities from 60 mA cm^−2^ to 160 mA cm^−2^ with an internal gap of 20 mA cm^−2^. Capacity-voltage profiles of VRFB single cell with several electrodes at (e) 60 mA cm^−2^ and (f) 160 mA cm^−2^. (g) Cycling performance of VRFB employing various electrodes at current density of 60 mA cm^−2^.

To verify the electrochemical performance in practical application of prepared CFs, the VRFB single-cell tests were conducted at various current densities from 60 to 160 mA cm^−2^ with an interval gap of 20 mA cm^−2^. Moreover, the measurements were performed with 1.5 M vanadium ions in 3 M H_2_SO_4_ at room temperature. The coulombic efficiency (CE), voltage efficiency (VE), energy efficiency (EE), and discharge capacity at various current densities are shown in Figure 3(a–d). All samples exhibited similar CE at the same current densities, although there were significant differences in VE, where ET-CF exhibited the best performance, which can be explained by the galvanostatic charge and discharge voltage profiles at a constant current density (Figure 3(e–f) and Figure S9). These results are primarily attributed to the hydrophilic properties of ET-CF, as confirmed by the electrolyte droplet test and XPS analysis, with an increased number of oxygen functional groups. Furthermore, based on these improved efficiencies, VRFB single cell equipped ET-CF delivered the highest specific discharge capacity of 18.6 Ah L^−1^ at a current density of 60 mA cm^−2^ which is considerably higher than that of p-CF (15.3 Ah L^−1^) and HT-CF (17.2 Ah L^−1^) resulting in significant electrolyte utilization (Figure S9) [39]. Note that even under an elevated current density of 160 mA cm^−2^, the VRFB single cell utilizing ET-CF exhibits 1.7 times greater specific capacity than that utilizing p-CF. This notable improvement can be attributed to the porous structure of ET-CF, which includes enhanced mass transportation of vanadium ions, thus providing a reasonable explanation for the remarkable electrochemical performance [29,30]. For the long-cycle durability test, the VRFB cell with HT-CF underwent a severe efficiency drop owing to the oxygen functional groups on the planar surface, which are easily degraded during cell operation [40,41]. However, in the case of ET-CF, the VRFB single cell operated without efficiency decay over 200 cycles at a current density of 60 mA cm^−2^, indicating high reversibility, which can be attributed to the robust oxygen functional groups at the graphite edge sites of ET-CF and its porous structure (Figure 3(g) and Figure S11).

## 4. Conclusion

In this study, we successfully fabricated a nanosized holey electrode (ET-CF) to improve the electrochemical performance of VRFB. Enhanced redox kinetics in both the catholyte and anolyte, even in a single cell with a lower overpotential, could be achieved by a *two-in-one* strategy to improve both the chemical and structural properties of the CF through Ni metal etching. From a chemical perspective, the formation of a porous structure on CF with abundant graphite edge sites, where oxygen-containing functional groups are prone to playing a crucial role in chemically offering more active sites to facilitate the redox reaction of vanadium ions. From a structural viewpoint, an increased specific surface area and a porous structure enables fast mass transfer through the active space. Owing to the synergetic effect of improved chemical and mechanical properties, the application of ET-CF to single-cell VRFB allows for achieving a high energy efficiency of 86.2% at current density of 60 mA cm^−2^ with ultra-stable cell operation without efficiency drop. Furthermore, even at a high current density of 160 mA cm^−2^, the VRFB single cell with ET-CF still has a superior energy efficiency of 72.2%. As a result, we believe that this study highlights the importance of robust graphite edge-sited oxygen functional groups and the holey structure of CF, emphasizing that high-efficiency VRFB can be achieved by engineering both the structure and surface properties of the CF electrode.

## Supplementary Material

Supplemental Material
