# Supplementary material for: Two-in-one strategy for optimizing chemical and structural properties of carbon felt electrodes for vanadium redox flow batteries
Source: Sci Technol Adv Mater. 2024 Mar 6;25(1):2327274. doi: 10.1080/14686996.2024.2327274 (PMC10962290; doi:10.1080/14686996.2024.2327274)
Supplement: Supplemental Material [file TSTA_A_2327274_SM6848.docx]

Supporting Information

***Two-in-one* strategy for optimizing chemical and structural properties of carbon felt electrodes for vanadium redox flow batteries**

Sung Joon Park^a†^, Min Joo Hong^b†^, Ye Ji Ha^a^, Jeong-In Choi^c^, Ki Jae Kim^a,d^*

^a^ Department of Energy Science, Sungkyunkwan University, Suwon, Gyeonggi-do 16419, Republic of Korea

^b^ Department of Future Energy Engineering, Sungkyunkwan University, Suwon 16419, Republic of Korea.

^c^ Department of Energy Engineering, Konkuk University, Neungdong-ro 120, Gwangjin-gu, Seoul 05029, Republic of Korea

^d^ SKKU Institute of Energy Science and Technology (SIEST), Sungkyunkwan University, Suwon 16419, Republic of Korea.

*** E-mail: [kijaekim@skku.edu](mailto:kijaekim@skku.edu) (K. J. Kim)

^†^These authors contributed equally to this study.


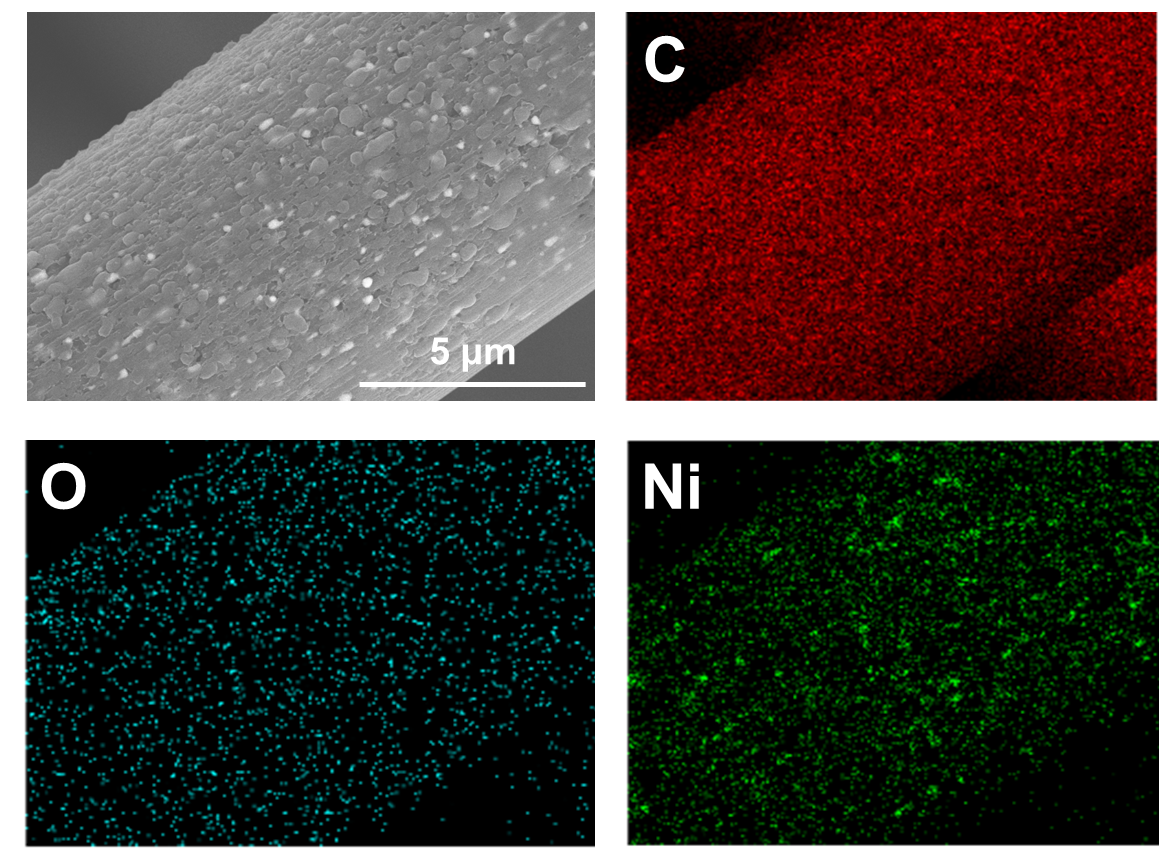


**Figure. S1**. SEM and EDS images of CF after stirring with nickel solution


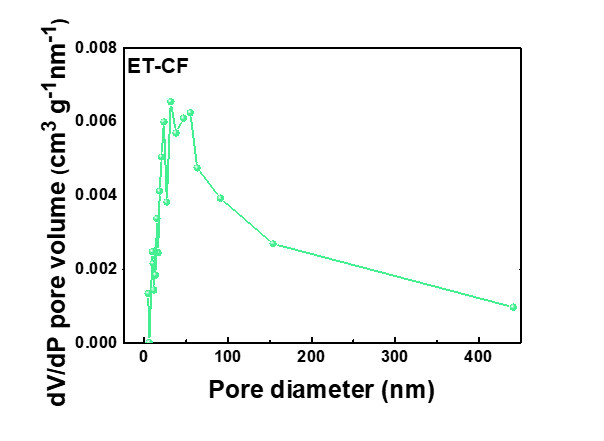


**Figure. S2**. Pore size distribution of ET-CF


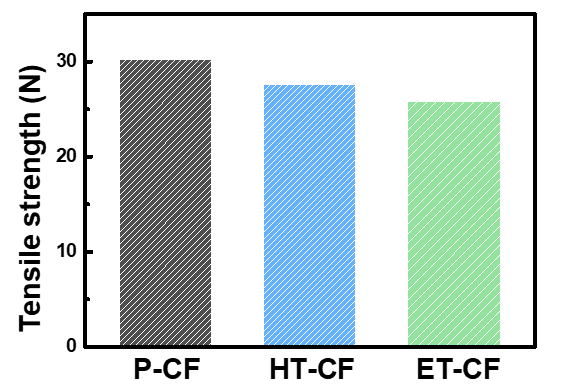


**Figure. S3**. Tensile strength of P-CF, HT-CF and ET-CF


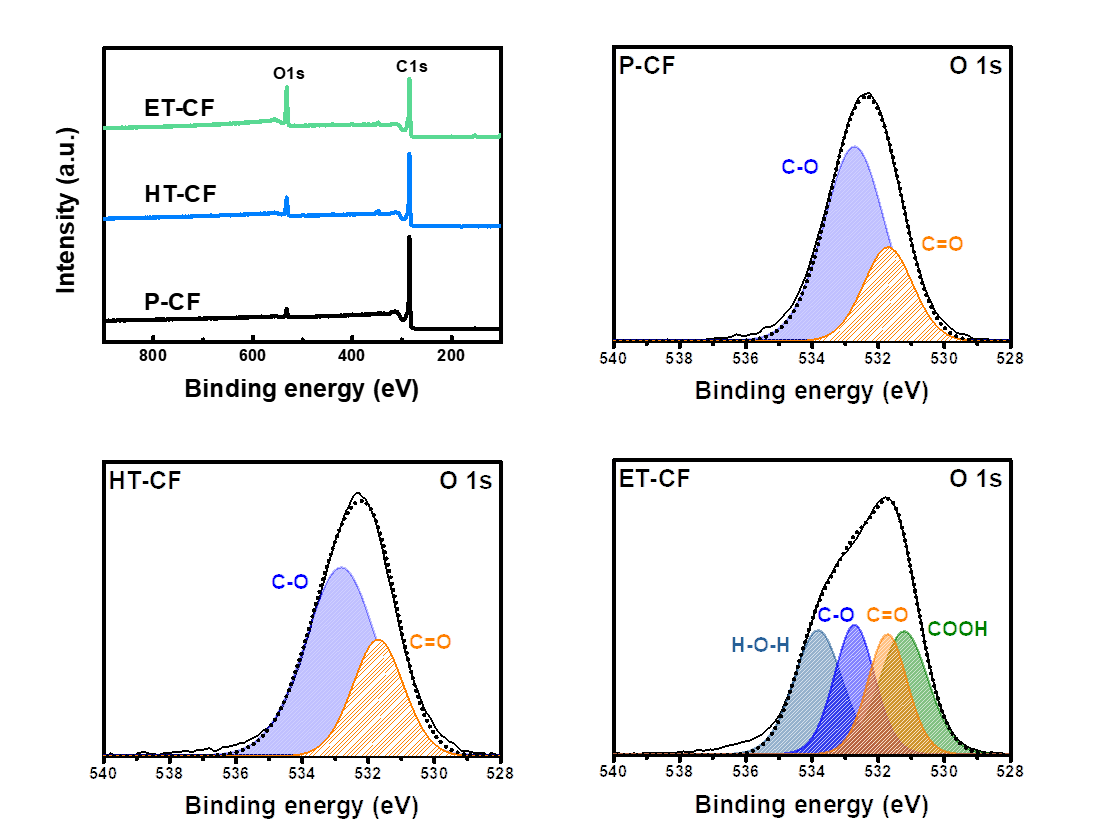


**Figure. S4**. XPS survey data and O 1s spectra of P-CF, HT-CF, and ET-CF respectively


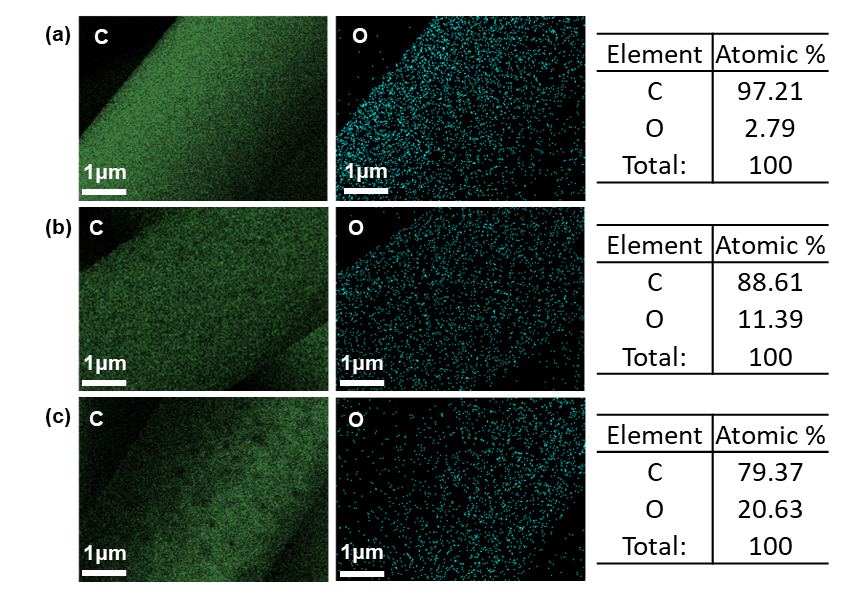


**Figure. S5**. EDS mapping images of (a) P-CF, (b) HT-CF, and (c) ET-CF


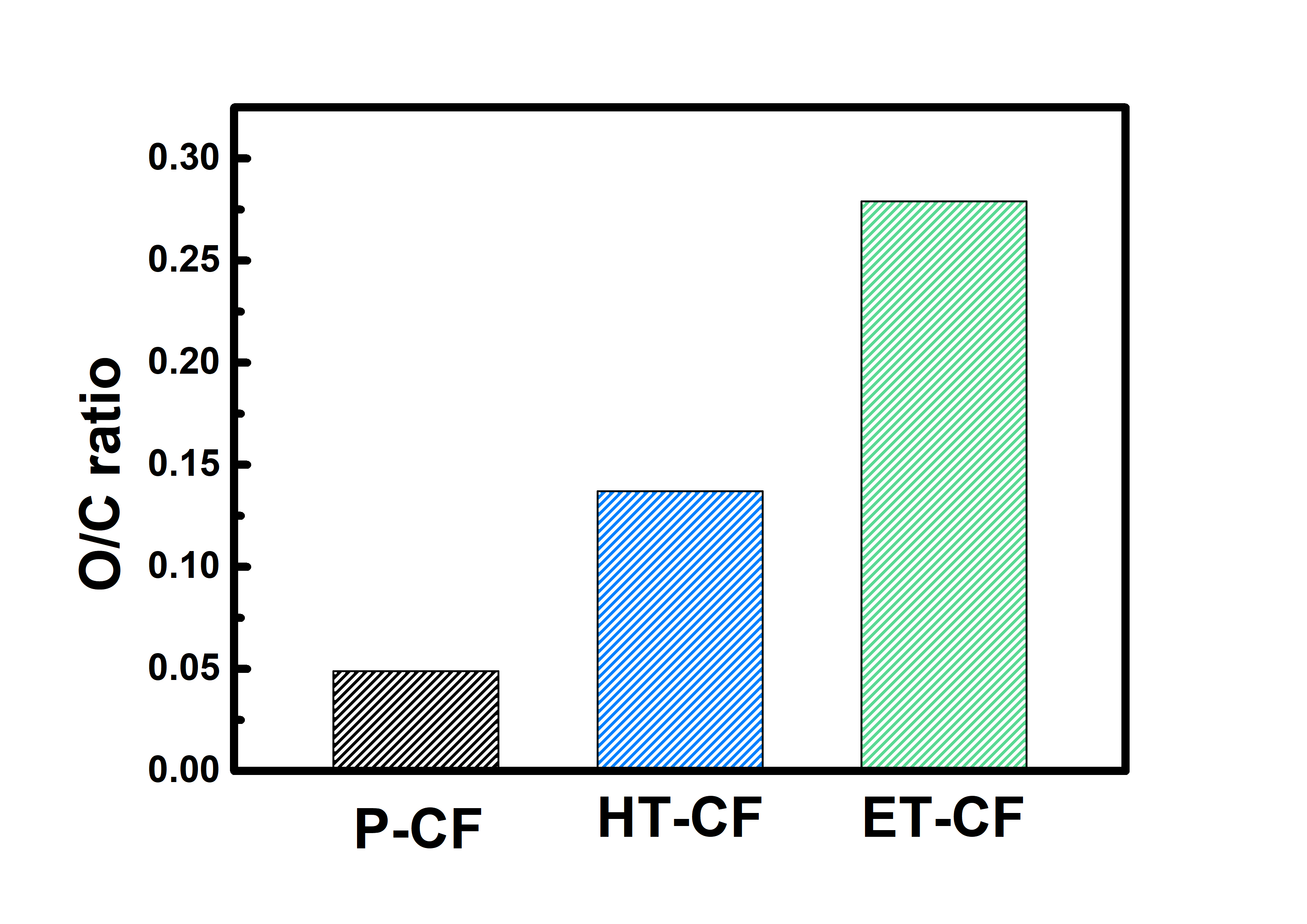


**Figure. S6**. O/C ratio of P-CF, HT-CF, and ET-CF


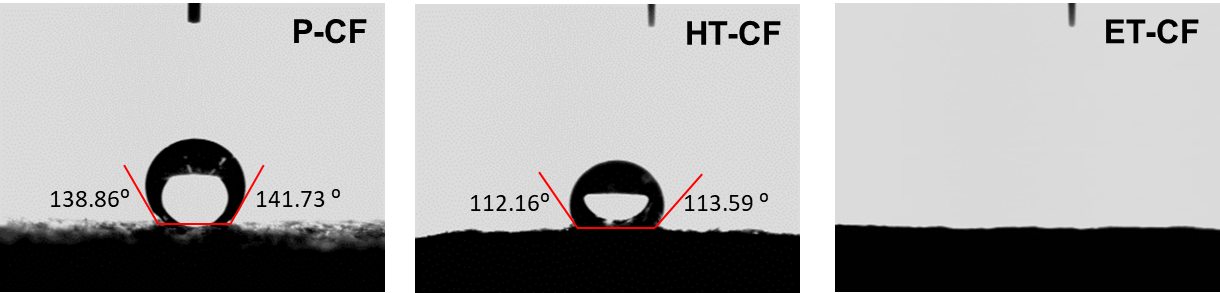


**Figure. S7**. Electrolyte droplet test of P-CF, HT-CF, and ET-CF


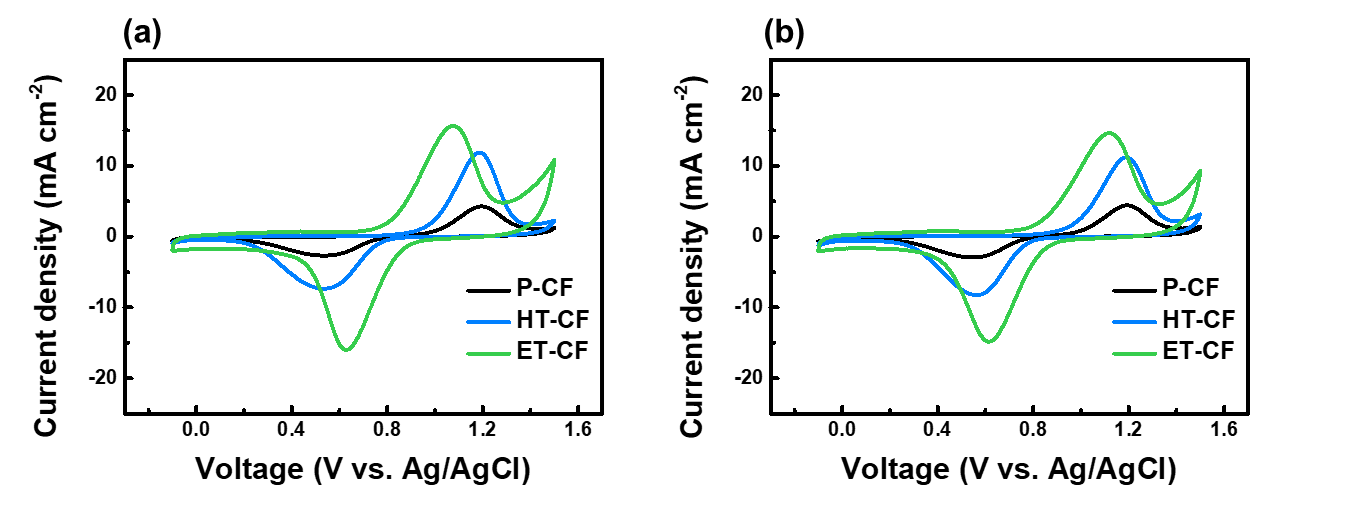


**Figure. S8**. (a) 50^th^ cycle and (b) 100th cycle of CV test at scan rate of 10 mV s^-1^


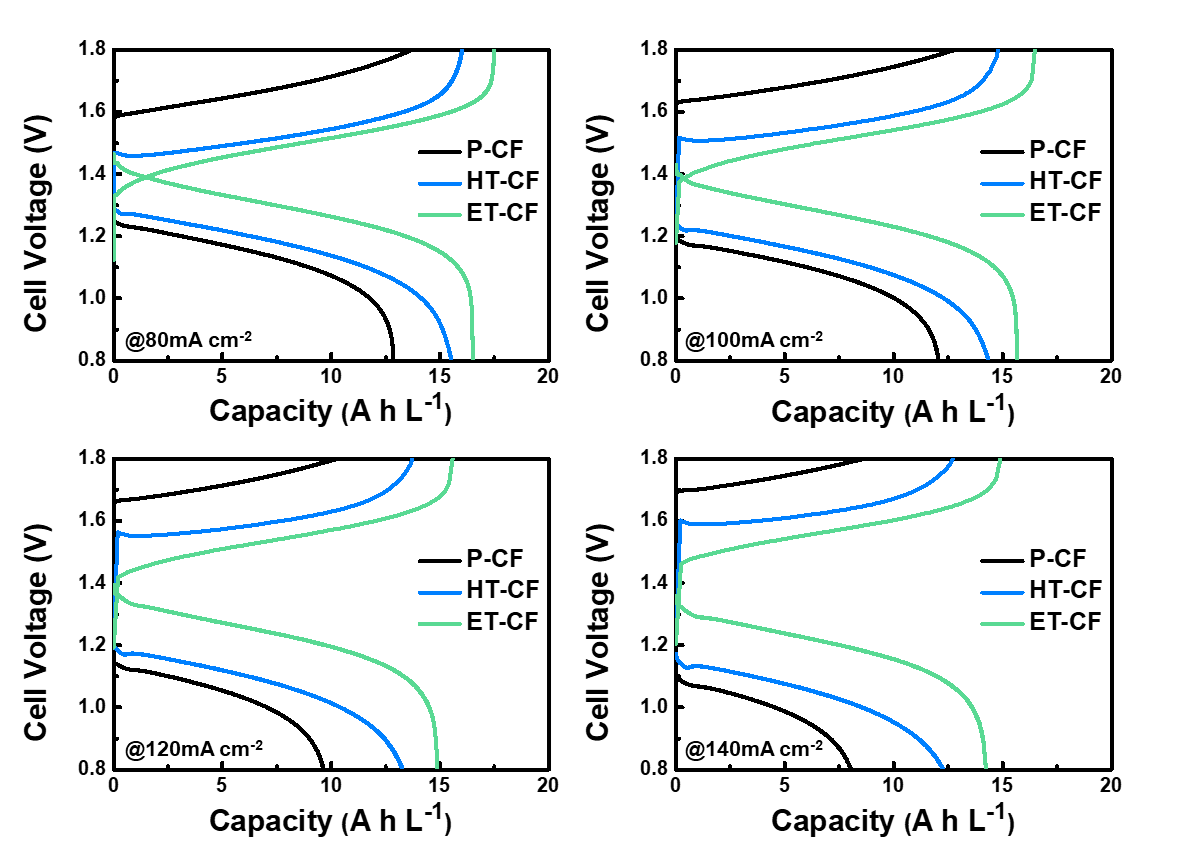


**Figure. S9**. Voltage profiles of VRFB with P-CF, HT-CF and ET-CF at 80, 100, 120, 140 mA cm^-2^. respectively


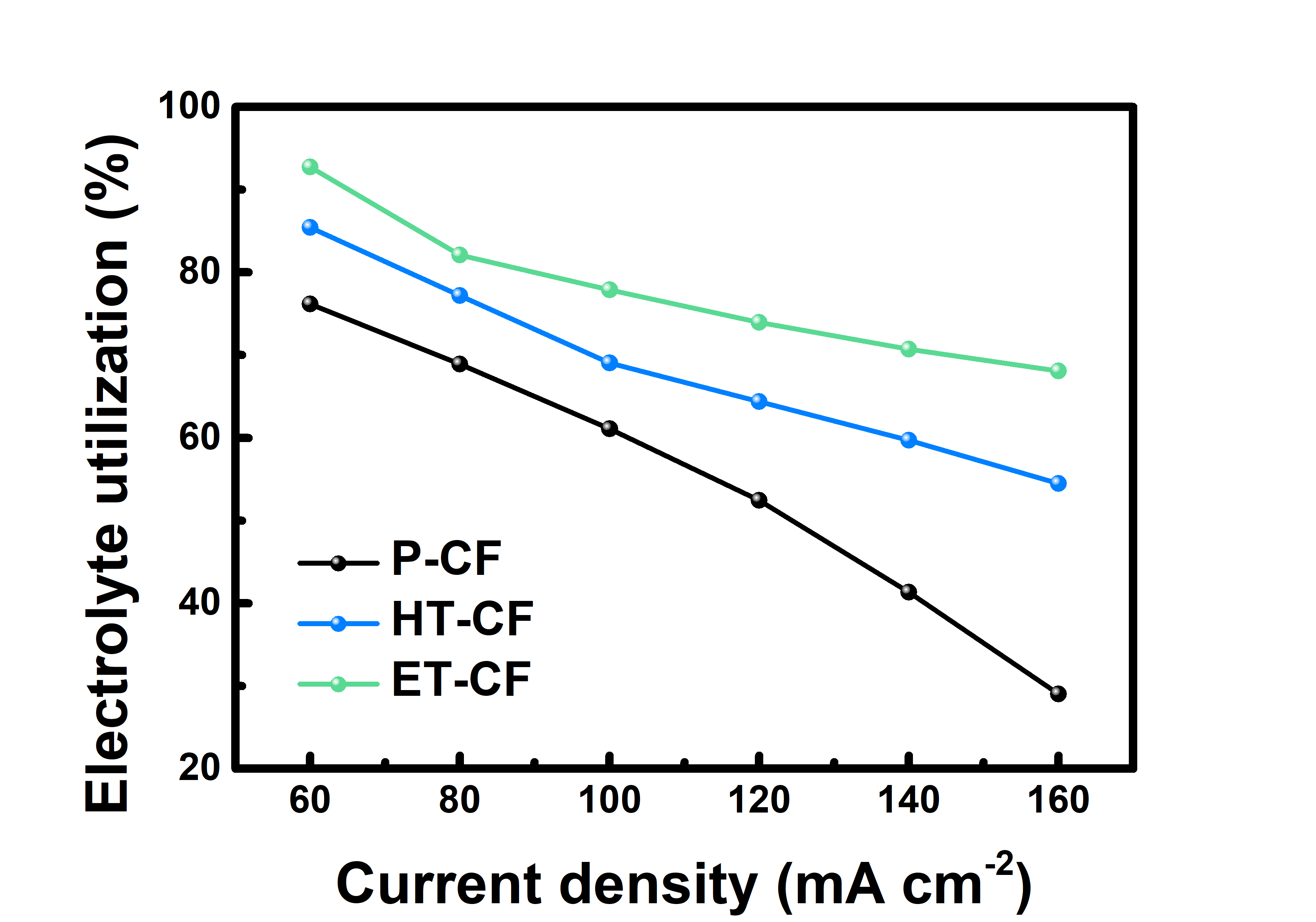


**Figure. S10**. Electrolyte utilization of P-CF, HT-CF, and ET-CF at various current densities from 60 to 160 mA cm^-2^


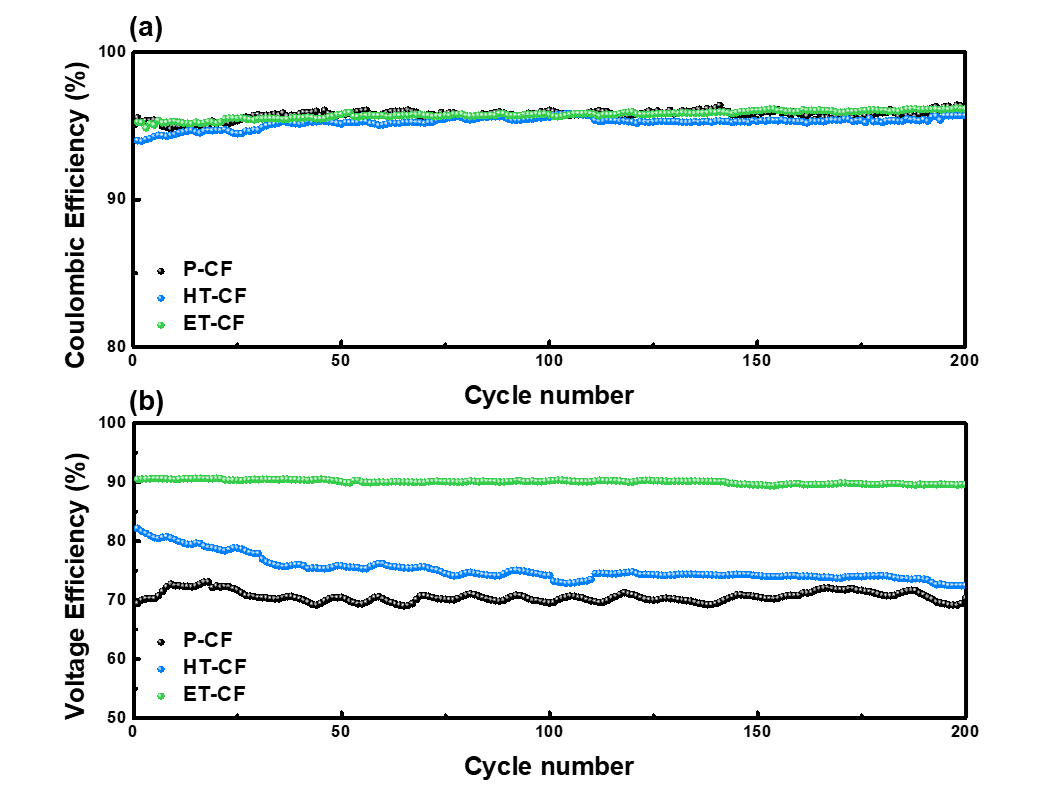


**Figure. S11**. (a) Coulombic efficiency and (b) Voltage efficiencies of the VRFB single cells measured for 200 cycles at current density of 60 mA cm^-2^.

**Table S1**. Parameters obtained from CV test

| 10 mV | I_pa_ (mA) | I_pc_ (mA) | \|I_pa_/I_pc_\| | E_pa_ (V) | E_pc_ (V) | ∆E (V) |
| --- | --- | --- | --- | --- | --- | --- |
| **Catholyte** | | | | | | |
| **P-CF** | 4.28 | -1.80 | 2.38 | 1.09 | 0.59 | 0.50 |
| **HT-CF** | 13.83 | -9.48 | 1.46 | 1.08 | 0.61 | 0.47 |
| **ET-CF** | 21.3 | -21.5 | 0.99 | 0.97 | 0.65 | 0.32 |
| **Anolyte** | | | | | | |
| **P-CF** | 6.15 | - | - | -0.22 | - | - |
| **HT-CF** | 6.63 | - | - | -0.32 | - | - |
| **ET-CF** | 15.5 | -28.64 | 0.54 | -0.42 | -0.61 | 0.19 |

**Table S2**. Slope of Randles-Sevcik plots

|  | **Oxidation slope** | **Reduction slope** |  | **Oxidation slope** |
| --- | --- | --- | --- | --- |
| **Catholyte** | | | **Anolyte** | |
| **P-CF** | 4.54 | 2.62 | **P-CF** | 1.05 |
| **HT-CF** | 7.62 | 4.26 | **HT-CF** | 10.40 |
| **ET-CF** | 11.01 | 9.69 | **ET-CF** | 12.09 |
